# Root dynamic growth strategies in response to salinity

**DOI:** 10.1111/pce.14205

**Published:** 2021-11-17

**Authors:** Yutao Zou, Yanxia Zhang, Christa Testerink

**Affiliations:** ^1^ Laboratory of Plant Physiology, Plant Sciences Group Wageningen University and Research Wageningen the Netherlands

**Keywords:** development, genome‐wide association studies (GWAS), gravitropism, growth, salinity

## Abstract

Increasing soil salinization largely impacts crop yield worldwide. To deal with salinity stress, plants exhibit an array of responses, including root system architecture remodelling. Here, we review recent progress in physiological, developmental and cellular mechanisms of root growth responses to salinity. Most recent research in modulation of root branching, root tropisms, as well as in root cell wall modifications under salinity stress, is discussed in the context of the contribution of these responses to overall plant performance. We highlight the power of natural variation approaches revealing novel potential pathways responsible for differences in root salt stress responses. Together, these new findings promote our understanding of how salt shapes the root phenotype, which may provide potential avenues for engineering crops with better yield and survival in saline soils.

## Abstract 2

This review highlights recent research on the effect of soil salinity on root growth modulation. We discuss root architecture plasticity, root tropisms and root cell wall modifications under salinity stress in *Arabidopsis thaliana* and several crop species.

## 1 INTRODUCTION

Increased soil salinity is a worldwide problem that causes yield loss of crops. Due to the current rising sodium chloride levels in groundwater, secondary salinization affects irrigated land, eventually leading to the loss of agricultural soils (FAO, 2015). Unlike halophyte species that are highly tolerant to salt concentrations (at least 200 mM NaCl), glycophyte plant species, that include most of our crops, can only grow healthily in low concentrations of salt (Cheeseman, 2015; Flowers & Colmer, 2008). Thus, research efforts aim to find physiological and genetic solutions to minimize the impact of saline land on global crop yield (Ismail & Horie, 2017; Munns et al., 2020; Munns & Gilliham, 2015). Salt stress is defined as the detrimental effect of high concentrations of salt accumulated in the soil, leading to inhibition of plant growth and development (Rahman, Ijaz, Qamar, Bukhari, & Malik, 2018). In saline soils, plants experience both osmotic and ionic stress. Osmotic stress arises due to the increased sodium ions in the soil, which leads to the reduction of water absorption, affecting various downstream processes in plants within several hours (Awlia, Alshareef, Saber, et al., 2021; Julkowska & Testerink, 2015). The ionic stress component was believed to affect plants much later after perception of the Na^+^ stimulus; when toxic NaCl levels were reached in the root and shoot tissues (Munns & Tester, 2008). However, recent studies show that plants can generate rapid ionic stress‐specific Ca^2+^ signals within 30 s (Choi, Toyota, Kim, Hilleary, & Gilroy, 2014; Jiang et al., 2019) and show internalization of PIN2 auxin transporters within an hour as a specific response to Na^+^ ions (Galvan‐Ampudia et al., 2013). Cellular signal transduction pathways induced upon salinity include calcium and cyclic guanosine monophosphate signalling, phospholipid signalling and reactive oxygen species (ROS) formation as well as protein kinase activation (Lamers, van Meer, & Testerink, 2020; van Zelm, Zhang, & Testerink, 2020). Moreover, cellular Na^+^/K^+^ balance modulated via ion transporters such as potassium channels and (anti‐) transporters also contribute to plant local and distal salt responses (van Zelm et al., 2020; Y. Yang & Guo, 2018). Together, these cellular processes affect plant growth and survival in salt.

Roots are able to sense multiple environmental stimuli, coordinate cellular stress responses and reorient their growth direction as reviewed (Lamers et al., 2020; Muthert, Izzo, van Zanten, & Aronne, 2020). Root growth strategies are dynamically changed in response to biotic and abiotic stresses, by modulation of specific key traits, such as root length and branching, redirection of root growth and modification of cell wall compositions. In salinized conditions, plants exhibit root phenotypic plasticity by modulating both root system architecture (RSA) components and directional growth dynamically (Dinneny, 2019; Julkowska et al., 2017; Korver et al., 2020). Understanding these dynamic root growth strategies and their underlying physiological, developmental and cellular mechanisms is expected to contribute to future agricultural strategies to improve crop yield in saline soils.

This review provides an overview of root architectural plasticity in responses to salinity stress and links new findings on mechanisms to phenotypes. We firstly discuss the basics of root phenotypes under default conditions without salt, and then compare to salinity conditions focusing on the physiological responses, root developmental growth, genetic and natural variation approaches and cell wall modulation.

## 2 RSA REMODELING STRATEGIES TO COPE WITH SALINITY

### 2.1 RSA: basic strategies to shape the root

The soil environment is variable and complex, thus to grow in soil, the development of roots needs to be flexible in response to many cues (Koevoets, Venema, Elzenga, & Testerink, 2016; Rellán‐Álvarez, Lobet, & Dinneny, 2016). The RSA of a plant describes the spatial configuration of the whole root system, that exhibits a considerable diversity among species, genotypes, space and time (Lynch, 1995). Monocotyledons and dicotyledons show fundamentally distinct patterns in RSA, as reviewed (Osmont, Sibout, & Hardtke, 2007). In general, RSA, as a plastic trait, presents the root 3D developmental plasticity that is established to avoid unfavourable environments and optimize the utilization of resources (Morris et al., 2017). One of the limitations is the difficulty in visualizing the root systems in natural soil situations because they are hidden underground. Recently, with the progress in technological development and mathematical concept applications, more visualization technologies become available, including the X‐ray computed tomography (CT), Growth and Luminescence Observatory for Roots (GLO‐Roots), soil rhizotron phenotyping and computational analysis approaches (Atkinson, Pound, Bennett, & Wells, 2019; Gandullo, Ahmad, Darwish, Karlova, & Testerink, 2021; Pagès, Pointurier, Moreau, Voisin, & Colbach, 2020; Rellán‐Álvarez et al., 2015; Teramoto et al., 2020; Yoshino et al., 2019). Yet, currently most of our knowledge on RSA plasticity and the genetic factors that control it, is still based on 2D systems, in particular of roots growing on agar media in vertical plates.

### 2.2 Salt modulates RSA

**Figure 1:**
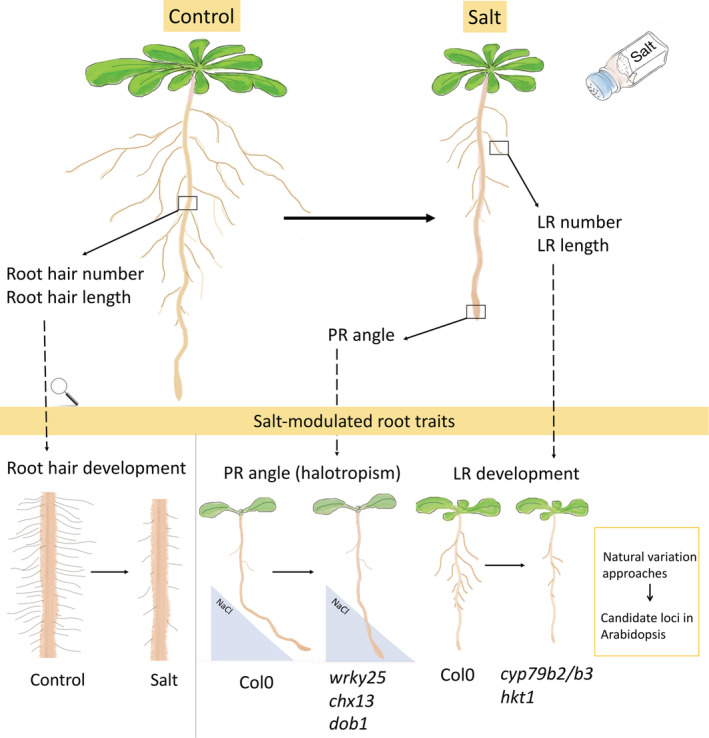
Root system architecture (RSA) remodelling in response to salt treatment. Salt modulates root shape through PR, LR and root hairs. Salt affects PR and root hair development by reducing both number and length, and specifically induces changes in PR growth angle in a halotropism assay. Natural variation approaches have led to the identification of several new genetic loci contributing to RSA changes in salt. The transcription factor *WRKY DNA‐BINDING PROTEIN 25 (WRKY25)*, *cation‐proton exchanger (CHX13)* and *Double Bending 1* (*DOB1*) were identified to be required for the salt avoidance (halotropism) response (Deolu‐Ajayi et al., 2019); *High‐affinity K* ^*+*^ *transporter 1 (HKT1)* and *CYTOCHROME P450 FAMILY 79 SUBFAMILY B2 (CYP79B2)* were identified to be involved in LRs development under salt stress (Julkowska et al., 2017) [Colour figure can be viewed at wileyonlinelibrary.com]

Plants exhibit notable changes in RSA in response to salt. The effect of salt treatment on root growth is largely dependent on the severity of the salt treatment. In wheat, salt inhibits root length in a dose‐dependent manner ranging from 50 to 200 mM NaCl (Rahnama, Munns, Poustini, & Watt, 2011). In *Arabidopsis*, moderate to high salt concentrations (75–150 mM NaCl) inhibit both primary root (PR) and lateral root (LR) growth (Figure 1). The traits of PR length, LR length and LR number are consistently reduced, but LR density shows a large variation in response to high salt concentrations between different reports, likely caused by different nutrient concentrations used (Julkowska et al., 2014; P. Li, Yang, et al., 2021; Y. Zhao, Wang, Zhang, & Li, 2011; Zolla, Heimer, & Barak, 2010). Under even higher salt concentrations (NaCl ≥ 200 mM), the emerging and young LRs (<100 μm) exhibit dramatically less damage, as compared with the PR and elongated LRs (> 400 μm) in viability assays (Ambastha, Friedmann, & Leshem, 2020). The difference observed could be due to the higher level of NADPH oxidase‐activated ROS induction in the young LR as compared to the PR (Ambastha et al., 2020). On the other hand, the relevance of these observations remains to be established, as they occur on a salt concentration that is lethal to the plants. Interestingly, low salt concentrations (<NaCl 50 mM) promote LR growth when 4 days or 5 days‐old seedlings are transferred to the NaCl stress medium (Julkowska et al., 2014; Zolla et al., 2010). Additionally, this effect was shown to be specific to salt stress, and did not occur in response to osmotic stress (Zolla et al., 2010). When seeds are germinated directly on the low‐stress medium of 30 mM NaCl, no difference in LR development was found (Y. Zhao et al., 2011), suggesting that the developmental stage is a crucial factor for the plant to activate different root responses to salinity via various molecular, cellular and physiological mechanisms. However, the mechanisms underlying the dynamic modulation of RSA during the different stages of plant development are still largely unclear.

Abscisic acid (ABA), as a general stress‐induced hormone, has a crucial role in regulating the plant growth response under saline conditions as reviewed (B. Li, Tester, & Gilliham, 2017; van Zelm et al., 2020). Salt stress induces a quiescent phase in LR outgrowth, which is mediated by the action of the ABA (L. Duan et al., 2013). Mutants of ABA‐independent class I SnRK2 members showed a reduction in PR length and LR density (Kawa et al., 2020; McLoughlin et al., 2012). How ABA‐dependent and ABA‐independent pathways together contribute to the effect of moderate (at least sub‐lethal) salt stress in modulating RSA at different developmental stages would be an interesting topic to be further investigated.

Root hairs are cylindrical extensions of epidermal cells that are an important aspect of RSA, contributing to water and nutrient uptake by increasing the absorptive surface area of the root (Correa, Postma, Watt, & Wojciechowski, 2019). Root hairs also enhance plant survival by promoting the root anchoring and water preservation capacity in soil, due to their tensile strength (Bengough Glyn, Loades, & McKenzie, 2016; Choi & Cho, 2019; C. Zhang et al., 2018). Multiple key genes have been reported to be involved in the developmental program of root hairs from initiation to outgrowth, including gene families of root hair defective and root hair defective six‐like (Han et al., 2020; Salazar‐Henao, Vélez‐Bermúdez, & Schmidt, 2016; Stanislas & Jaillais, 2019). When exposed to salt, the number of root hairs is dramatically reduced, which could be partially attributed to the large decrease in epidermal cell numbers under salt stress (Dinneny, Long, Wang, et al., 2008; Y. Wang et al., 2008). Notably, ionic stresses (NaCl, KCl or LiCl) could reduce both root hair density and elongation, while an equi‐osmolar concentration of mannitol increased both root hair density and elongation, indicating different mechanisms in regulating root hair growth between ionic and osmotic stresses (Y. Wang et al., 2008). In response to salt treatment, both cell autonomous and non‐autonomous effects were observed by transcriptional profiling of three root hair epidermal patterning mutants. Among the identified salt‐responsive genes, many of these genes repressed by salt were involved in cell wall structure and trichoblast differentiation (Dinneny et al., 2008). Interestingly, salt inhibited the root hair outgrowth immediately after the treatment but then resumed after 8 hr, indicating the root hair responses are dynamic (Dinneny et al., 2008). However, current knowledge is still very limited on root hair developmental regulation and its contribution to salt stress responses.

## 3 ROOT TROPISMS DURING SALINITY STRESS: HOW TO GROW DOWN AND HOW TO AVOID SALT

Plant roots are capable to reorient their growth direction towards or away from specific stimuli, exhibiting gravitropism (Morita & Tasaka, 2004; Su, Gibbs, Jancewicz, & Masson, 2017), halotropism (Galvan‐Ampudia et al., 2013; Korver et al., 2020), hydrotropism (Dietrich et al., 2017; Eapen, Barroso, Ponce, Campos, & Cassab, 2005), phototropism (Kimura et al., 2018; Silva‐Navas et al., 2016), chemotropism (Ferrieri et al., 2017; Kellermeier et al., 2014) and thigmotropism (Massa & Gilroy, 2003). Under normal and mildly saline conditions, gravitropism plays an overwhelming and continuous role in modulating root growth direction. The plant hormone auxin mediates both gravity sensing and subsequent responses (Y. Zhang, He, et al., 2019). After gravitropic signal integration, auxin is transported and accumulated at the lower side of the root elongation zone of a horizontally placed root, where it rapidly inhibits root cell elongation (Abas et al., 2006; Brunoud et al., 2012; Fendrych et al., 2014; Fendrych, Leung, & Friml, 2016). Auxin redistribution largely depends on the auxin importers AUXIN/LIKE‐AUX1 (AUX/LAX) and the PIN‐FORMED (PIN) family of auxin efflux carriers, namely PIN1, PIN2, PIN3, PIN4 and PIN7‐work together to establish a root “auxin reflux loop” as summarized in reviews (Adamowski & Friml, 2015; Korver, Koevoets, & Testerink, 2018; Zhou & Luo, 2018).

**Figure 2:**
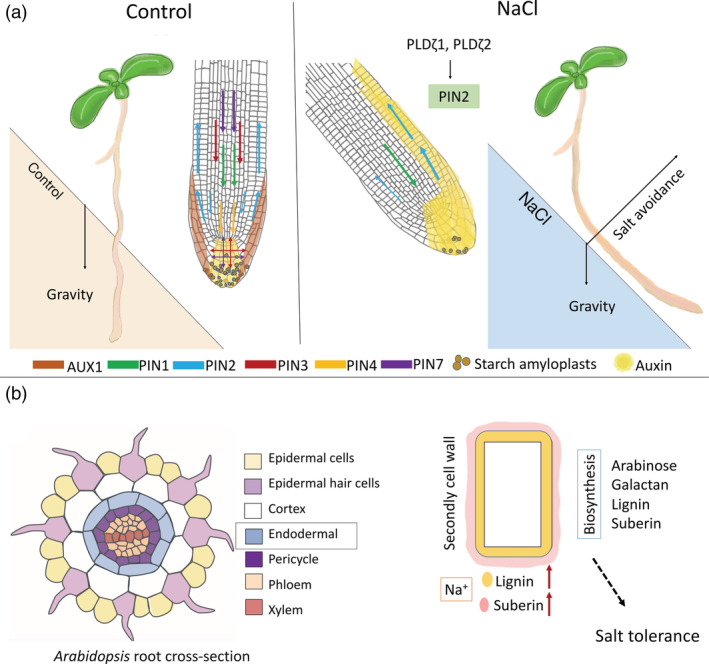
Root cellular responses to salt. (a) Roots find the balance between tropisms to avoid salt. The directional growth depends on the dynamic auxin reflux loop with the involvement of the auxin importer AUX1 and the PIN‐FORMED (PIN) family. Root growth follows the gravity vector under normal conditions, while under salt conditions, roots exhibit a salt avoidance response by redistribution of auxin in the root tip, which is dependent on the ζ‐type PLDs (PLDζ1 and PLDζ2) that mediate the re‐localization of PIN2. Only the changed PINs were shown for salt treatment. (b) Cell wall modulations under salt treatment. Salt induces cell wall composition changes, including the increased deposition of lignin and suberin. In *Arabidopsis*, salt‐induced lignification and suberization mainly occur at the root endodermal layer, as exodermis does not exist in *Arabidopsis* roots; while in other species (rice and maize), it occurs at both endodermal and exodermal root layers. Biosynthesis of arabinose, galactan, lignin and suberin, can contribute to plant salt tolerance. Cartoon of root tissue cells adapted with permission (Bouche, Frederic [2017]: Arabidopsis—Root cell types. Figshare. https://doi.org/10.6084/m9.figshare.4688752.v2.) [Colour figure can be viewed at wileyonlinelibrary.com]

Under high salinity, plants can reduce their exposure to salinity by changing their roots' growth direction to avoid a saline environment through a response called halotropism. Not only the roots of *Arabidopsis*, but also the roots of tomato (*Solanum lycopersicum*) and sorghum (*Sorghum bicolor*) seedlings, either on agar media or in soil, are able to exhibit a halotropic response when exposed to a salt gradient (Galvan‐Ampudia et al., 2013). Halotropism is accomplished by redistribution of auxin in the root tip mainly by the PIN2 auxin efflux carrier. When exposed to a salt gradient, salt triggers internalization of PIN2 at the saltier side of the root, consequently leading to auxin redistribution and the directional bending away from salt (Galvan‐Ampudia et al., 2013). In addition, other auxin transporters are also relevant to halotropism. For example, a recent study showed that salt induces the transient upregulation of PIN1 in the stele, and an elevation of AUX1 levels on the non‐salt‐exposed side (van den Berg, Korver, Testerink, & ten Tusscher, 2016). In accordance, *pin1* mutants show a delayed halotropic response, demonstrating a role for PIN1 in halotropism (van den Berg et al., 2016; Figure 2).

Notably, it was shown that osmotic stresses (both NaCl and mannitol treatments), immediately enhance clathrin‐mediated endocytosis, increase PIN endocytosis and overcome the inhibitory effect of auxin (Baral et al., 2015; Nakayama et al., 2012; Zwiewka, Nodzyński, Robert, Vanneste, & Friml, 2015). Moreover, a recent study showed that both NaCl and sorbitol treatments induce large membrane structures at the plasma membrane in epidermal and LR cap cells (Korver et al., 2020). In response to both ionic and osmotic stresses, there is a crucial role for ζ‐type PLD (PLDζ1) that is required for the relocalization of auxin carrier PIN2, one of the major factors in halotropism (Korver et al., 2020). On the other hand, despite the overlap with osmotic responses, roots also seem to exhibit a NaCl‐specific cellular response as a NaCl gradient affected PIN2 subcellular localization in epidermal cells while mannitol did not affect PIN2 localization, and also the observed phenotypic response is specific to NaCl and not induced by KCl or osmotic stress (sorbitol or mannitol) gradients at equivalent concentrations (Deolu‐Ajayi et al., 2019; Galvan‐Ampudia et al., 2013). In accordance, the PIN2‐GFP signal on the lateral side of the membrane of epidermal cells was not affected by sorbitol in either Col‐0 or a *pldζ1* mutant, but was induced by NaCl in Col‐0. This response was absent in the *pldζ1* mutant, indicating that lateral PIN2 re‐localization is specific to salt and depends on PLDζ1 function (Korver et al., 2020; Figure 2). In comparison, hydrotropism, a response of roots toward water, often also assessed on sorbitol gradients, only manifests itself typically at much higher osmotic stress levels (Dietrich et al., 2017). Yet, how the observed cellular responses contribute to the salt‐specificity of the physiological halotropic response, remains to be elucidated.

Under saline conditions, gravitropic growth is attenuated by degradation of amyloplasts in root columella cells, which is correlated with PIN2 transcription level and leading to auxin distribution in salt stress (Sun et al., 2008). The inhibition of root gravitropic growth by salt is dose‐dependent (from 0 to 150 mM; Sun et al., 2008). In addition, on salt gradients, roots firstly display salt avoidance but eventually follow the gravitropic response at lower salt concentrations, while at high salt roots are able to overcome gravitropism and continue to exhibit halotropism (Galvan‐Ampudia et al., 2013), thus suggesting a quantitative decisive balancing act determined by the relative strength between these two tropisms. In addition, light has been suggested to play a role in mediating root halotropism. The turfgrass species rough bluegrass (*Poa trivialis L*.) exhibits enhanced halotropism in blue light compared with white light, while in the dark or red light rough bluegrass does not exbibit halotropism (Petrella et al., 2018). Therefore, it is indispensable to build up knowledge not only on single stimuli modulating tropisms, but also on tropism‐tropism interaction, and the mechanisms underlying the responses. In addition, while negative halotropism of glycophytes has been relatively well characterized, further investigations are still needed for a possible positive halotropic response of halophytes (Shelef, Lazarovitch, Rewald, Golan‐Goldhirsh, & Rachmilevitch, 2010). This may provide a better understanding on whether plants are capable of flexibly and dynamically governing the movement of their roots towards or away from salt, based on their preferred salt level.

## 4 UNCOVERING THE GENETIC CONTROL OF ROOT PLASTICITY

Natural variation studies have proven to be successful in identifying novel genetic components and uncovering relevant loci that are associated with agronomic traits. This approach relates phenotypic variation to genetic variation thereby statistically associating traits with sequence polymorphism in natural accessions (Alonso‐Blanco et al., 2016; Seren et al., 2013). Several novel root growth regulation factors identified via genome wide association studies (GWAS) were reported to be associated with RSA traits in both default (without salt) and saline conditions. In Arabidopsis, *Cytokinin oxidase 2 (CKX2)* was identified to be associated with a gravitropic setpoint angle of LRs. CKX2 affects LR directional growth and cellular elongation by controlling the metabolism of cytokinin (Waidmann, Ruiz Rosquete, Schöller, et al., 2019). Additionally, *Exocyst70A3* was identified to control the depth of the root system by modulating PIN4‐dependent auxin transport (Ogura et al., 2019). Both CKX2 and EXO70A3 modulate RSA components via auxin‐dependent root gravitropic growth, again showing a crucial role of auxin transport and signalling in root phenotypes.

The genetic loci encoding for the transcription factor WRKY DNA‐BINDING PROTEIN 25 (WRKY25), CATION‐PROTON EXCHANGER 13 (CHX13) and DOUBLE BENDING 1 (DOB1) were identified to be associated with the salt avoidance response of the PR in a halotropism assay. In addition, CHX13 and DOB1 regulate Na^+^/K^+^ homeostasis and shoot dry weight under salt stress (Deolu‐Ajayi et al., 2019). Expression of *High‐affinity K* ^*+*^ *transporter 1 (HKT1)* was identified to be associated with average LR length. High *HKT1* expression in the root stele inhibits LR development in salt, which can be partially rescued by adding potassium (Julkowska et al., 2017). Moreover, CYTOCHROME P450 FAMILY 79 SUBFAMILY B2 (CYP79B2) has an important role in biosynthesis of camalexin, indole glucosinolates and auxin, by converting tryptophan to indole‐3‐acetaldoxime (IAOx). *CYP79B2* was identified by GWAS to be associated with the ratio of average lateral and main root length. The *cyp79b2/cyp79b3* double mutants showed a reduction in both number and length of LRs under salt stress, but not under control conditions (Julkowska et al., 2017; Figure 1).

For crops, genetic control of RSA is largely unelucidated, while natural variation in RSA plasticity in response to salt has been found in maize and rice. In maize, the Aux/IAA TF family member *ZmIAA1* and the GRAS‐type TF family member *ZmGRAS43*, were identified to be associated with modulating RSA in response to salinity stress (P. Li, Yang, et al., 2021). Another recent study showed that rice yields in saline soil can be improved via a QTL containing *SOIL SURFACE ROOTING 1 (qSOR1)*, a homolog of *DEEPER ROOTING 1 (DRO1)*, which is modifying root growth direction to a shallower root growth angle (Kitomi et al., 2020). However, in the absence of ion accumulation data of the soil the physiological relevance of the benefit of developing shallower root systems in this case is still unclear. In contrast, tomato seedlings growing in rhizotrons placed their LRs preferentially lower, avoiding the higher soil layers in which salt accumulated (Gandullo et al., 2021). Here, the genetic loci that contribute to the response remain to be characterized. Thus, a better understanding of RSA modulation is required to directly contribute to salt tolerance and may provide guidance to future breeding in salinized soil.

## 5 CELL WALL COMPOSITION MODULATION UNDER SALT STRESS

Plant cells are surrounded by cell walls that typically consist of cellulose, hemicellulose, pectin, structural proteins, glycoproteins and lignin for some cell types. Salt induced cell wall responses, including polysaccharide deposition, pectin modifications and microfibril orientation benefit plants to survive in salt stress as reviewed (Byrt, Munns, Burton, Gilliham, & Wege, 2018). Cell wall composition can be changed dynamically in response to biotic and abiotic stresses, and differs between species and cell types (Feng, Lindner, Robbins, & Dinneny, 2016; Vaahtera, Schulz, & Hamann, 2019; Wolf, Hématy, & Höfte, 2012).

Recent studies show that biosynthesis genes of cell wall components, including lignin (A. Q. Duan et al., 2020), arabinose (C. Zhao et al., 2019) and galactan (Yan et al., 2021), can contribute to salt tolerance. The modulation of root structural barriers via cell wall composition changes could reduce water loss and limit Na^+^ entry in roots (Byrt et al., 2018). Such changes include lignification and suberization in both endodermis and exodermis (Barberon, Vermeer, De Bellis, et al., 2016; Kajala et al., 2021). Lignification in higher plants provides firmness and hydrophobicity to the secondary cell walls and thus forms a barrier to protect plant against stresses (Q. Zhao, 2016). Similarly, suberization also generates hydrophobic and lipophilic secondary cell wall macromolecules acting as a barrier to restrict water and solutes (Andersen, Barberon, & Geldner, 2015). Salt stress increases root lignin content while reducing arabinoxylan content in maize (Oliveira et al., 2020). In Arabidopsis, overexpression of a suberin biosynthesis enzyme β‐Ketoacyl‐CoA Synthase (VvKCS) from grape *Vitis vinifera L*. enhanced salt tolerance (Z. Yang et al., 2020). In rice, class II *TREHALOSE‐PHOSPHATE‐SYNTHASE* gene (*OsTPS8*) was identified to positively regulate suberin deposition and to enhance salt tolerance (Vishal, Krishnamurthy, Ramamoorthy, & Kumar, 2019).

The Casparian strip, primarily made of lignin, is affecting the radial transport of water and minerals to the vasculature tissue (Naseer et al., 2012). After exposure to salt, the radial width of the Casparian strip was increased in maize (Karahara, Ikeda, Kondo, & Uetake, 2004). In Arabidopsis, salt stress enhances suberization of the root endodermal layer (Barberon et al., 2016), while in rice and maize lignification and suberization occur at both endodermal and exodermal root layers (Krishnamurthy et al., 2009; Krishnamurthy, Ranathunge, Nayak, Schreiber, & Mathew, 2011; Tylová, Pecková, Blascheová, & Soukup, 2017). However, the underlying molecular mechanisms of how barriers at the root endodermis and exodermis affect plant salt stress responses remain unclear.

Salt‐induced cell wall dynamic modifications also induce cell wall integrity sensing pathways, monitored by cell wall localized receptor‐like kinases, such as FERONIA (FER), to induce downstream signalling and further regulate salt tolerance (Dünser et al., 2019; Feng et al., 2018; Gigli‐Bisceglia, van Zelm, Huo, Lamers, & Testerink, 2020). Together, these findings suggest that dynamic salt‐induced changes in root cell wall compositions, as well as cell wall sensing are crucial for plant salt responses and ultimately tolerance.

## 6 CONCLUSION AND PROSPECTS

Plants can dynamically adjust their root growth components (e.g., density, length and angle) locally in response to salinity, to minimize their metabolic cost and the impact of the stresses. Local root growth plasticity responses appear crucial for plants to optimize architecture spatially and temporally under stress conditions (Julkowska et al., 2017; Kazan & Lyons, 2016; Korver et al., 2018). However, the regulation of salt‐induced root growth plasticity is still largely unknown. The current available tools using plant tissue‐specific and cell‐specific approaches that can analyse a few cells or a small area specifically based on mass spectrometry, biosensors or single‐cell omics can help us to unravel the mechanisms underlying local root growth in response to salt stress (Chen et al., 2020; Novák, Napier, & Ljung, 2017). We speculate that by activating local stress response pathways to accomplish local growth plasticity, salt stress could induce not only local developmental changes, but also systemic or distal signals, which are also crucial for adequate stress responses (H. Li, Testerink, et al., 2021).

This review discussed the regulation of dynamic root growth by salt and the underlying mechanisms that are expected to contribute to future agricultural efforts to promote crop stress resilience. In a natural heterogeneous soil, environment roots need to flexibly respond to several environmental cues. Thus, plant root growth plasticity in saline soil presents the outcome of the root dynamic growth affected by salt stress, soil type, climate, biota and other environmental conditions. Further prediction and modelling for the complex interactions between roots and saline soil is required, to provide insights into the mechanisms and new leads for improving agricultural production.

Moreover, more research efforts are needed to expand the knowledge beyond *Arabidopsis*; the lack of molecular tools is hindering the study of different crop species and natural varieties. On the positive side, natural variation approaches have successfully identified new genetic components contributing to salinity tolerance in crops species, including rice (Patishtan, Hartley, de Carvalho, & Maathuis, 2018), maize (M. Zhang, Liang, et al., 2019), tomato (Z. Wang et al., 2020), soybean (W. Zhang, Liao, et al., 2019) and barley (*Hordeum vulgare*; Hazzouri et al., 2018), which will be valuable molecular entries to further characterize the molecular mechanisms and to contribute to future engineering of more resilient crops.

Finally, the progress in developing new phenotyping techniques and platforms will help us capture the diverse root local responses among different species in both spatial and temporal aspects, and their relevance for salt resilience. For example, different RSA patterns were shown to be correlated with shoot Na^+^/K^+^ homeostasis in Arabidopsis when they were grown in agar plates (Julkowska et al., 2014). In tomato plants grown in soil, the most severe change in RSA under salt conditions was the suppression of LR emergence at the soil surface were salts accumulate, which could be an adaptive response (Gandullo et al., 2021). On the other hand, in rice, the near‐isogenic line qsor1‐NIL that had ‘soil‐surface roots’ showed a significant increase in yield in saline paddies, compared to the cultivars without the soil‐surface roots (Kitomi et al., 2020). Therefore, further evidence on a range of species and soil conditions is needed before general or specific beneficial root response strategies can be identified that would be future target phenotypes for breeding to optimally support whole plant salt resilience.

## CONFLICT OF INTEREST

The authors declare that there is no conflict of interest.

## Data Availability

Data sharing not applicable to this article as no datasets were generated or analysed during the current study.
